# Combined fMRI Region- and Network-Analysis Reveal New Insights of Top-Down Modulation of Bottom-Up Processes in Auditory Laterality

**DOI:** 10.3389/fnbeh.2021.802319

**Published:** 2022-01-18

**Authors:** Katarzyna Kazimierczak, Alexander R. Craven, Lars Ersland, Karsten Specht, Magda L. Dumitru, Lydia B. Sandøy, Kenneth Hugdahl

**Affiliations:** ^1^Department of Biological and Medical Psychology, University of Bergen, Bergen, Norway; ^2^Mohn Medical Imaging and Visualization Centre, University of Bergen, Haukeland University Hospital, Bergen, Norway; ^3^Department of Clinical Engineering, Haukeland University Hospital, Bergen, Norway; ^4^Department of Education, The Arctic University of Norway UiT, Tromsø, Norway; ^5^Department of Radiology, Haukeland University Hospital, Bergen, Norway

**Keywords:** dichotic listening, fMRI, network connectivity, DMN, EMN, attention, executive control, bottom-up/top-down

## Abstract

Dichotic listening along with the right-ear advantage (REA) has been a standard method of investigating auditory laterality ever since it was first introduced into neuropsychology in the early 1960s. Beginning in the 1980s, authors reported that it was possible to modulate the bottom-up driven perceptual REA by instructing subjects to selectively attend to and report only from the right or left ear. In the present study, we investigated neuronal correlates of both the bottom-up and top-down modulation of the REA through two fMRI analysis approaches: a traditional region approach and a network connectivity approach. Blood-Oxygenation-Level-Dependent (BOLD) fMRI data were acquired while subjects performed the standard forced-attention paradigm. We asked two questions, could the behavioral REA be replicated in unique brain markers, and second if the profound instruction-induced modulation of the REA found in behavioral data would correspond to a similar modulation of brain activation, both region- and network-specific modulations. The subjects were 70 healthy adult right-handers, about half men and half women. fMRI data were acquired in a 3T MR scanner, and the behavioral results replicated previous findings with a REA in the non-forced (NF) and forced-right (FR) conditions, and a tendency for a left-ear advantage (LEA) in the FL-condition. The fMRI data showed unique activations in the speech perception areas of the left temporal lobe when directly contrasted with activations in the homologous right side. However, there were no remaining unique activations when the FR- and FL-conditions were contrasted against each other, and with the NF-condition, using a conservative significance thresholding. The fMRI results are conceptualized within a network connectivity frame of reference, especially with reference to the extrinsic mode network (EMN). The EMN is a generalized task-positive network that is upregulated whenever the task demands exceed a certain threshold irrespective of the specifics and demands of the task. This could explain the similarity of activations for the FR- and FL-conditions, despite the clear differences in behavior.

## Introduction

Dichotic listening (DL) has been a standard method for the study of perceptual laterality in the auditory domain, both in healthy subjects and clinical populations. Although a variety of paradigms has been applied over the years, a commonly used paradigm is the consonant-vowel (CV) syllables paradigm (see Bryden, 1988; Tervaniemi and Hugdahl, 2003; Hugdahl et al., 2009; Beste et al., 2018; Westerhausen and Samuelsen, 2020). This involves presentation through headphones of two CV syllables, one delivered to the right ear and the other simultaneously delivered to the left ear. Because of the anatomical wiring of the auditory perceptual system, the right ear syllable in a dichotic situation will initially be presented to the left auditory cortex, while the left ear syllable will initially be presented to the right auditory cortex (originally suggested by Kimura, 1967). The phonetic nature of a CV-syllable and the preference for the left hemisphere to make the initial decoding of a phonetic signal is the basis for the right-ear advantage (REA). This means that subjects under normal circumstances will report more correct syllables from the right compared to the left ear.

Although the REA is a valid and reliable marker of left hemisphere speech sound specialization (see also Kimura, 1961; Hugdahl and Hammar, 1997; Voyer, 1998; Tervaniemi and Hugdahl, 2003; Westerhausen, 2019), also cross-validated in electrophysiological and neuroimaging studies (Hugdahl et al., 1999; Brancucci et al., 2004; Thomsen et al., 2004a; van den Noort et al., 2008), it soon became apparent that the REA could be modulated and even shifted to a left-ear advantage (LEA) by instructing the subjects to explicitly focus and attend to only the right or left ear stimulus, originally shown by Bryden et al. (1983) and Hugdahl and Andersson (1986). Thus, it was evident that the REA was subject to higher cognitive influences, in particular attention and executive functions (see also Hiscock et al., 1999; Hugdahl et al., 2009; for reviews, Cacace and McFarland, 2013; Westerhausen et al., 2013). Hugdahl and Andersson (1986) labeled this variant of the standard CV-syllables paradigm the “forced-attention DL paradigm” which means that the set of CV-syllables are presented three times in a row. The first condition is called “non-forced” (NF) and contains no instructions about focus of attention. In the next two conditions, the subject is explicitly instructed “to only listen to and focus on what you hear in the right (or left) ear,” with the right and left ear instructions being randomly shifted across subjects. The two instructed conditions are labeled “forced-right” (FR) and “forced-left” (FL), respectively (see Hugdahl and Andersson, 1986). What typically happens is that the REA is increased in the FR-condition compared to the NF-condition, while it is decreased or even shifted to a LEA in the FL-condition.

Taking the REA in the NF-condition as a baseline against which to evaluate the change in the FR- and FL-conditions, it is suggested that the REA in the NF-condition reflects a bottom-up perceptually driven laterality effect, while the REA in the FR-condition, in addition, reflects the effect of a top-down attentional effect. Thus, in the FR-condition bottom-up and top-down forces would act agonistically, or additive, to produce an increase in the magnitude of the REA. In the FL-condition the two forces would act antagonistically, or subtractive, which would reduce the magnitude of the REA, or even shifting it to a LEA. The reason for this is a processing conflict in the FL-condition, where the in-built bottom-up laterality effect pushes for processing of the right-ear stimulus, while the top-down instruction-effect pushes for processing of the left-ear stimulus, and cognition overrides a perceptual laterality effect (see Løberg et al., 1999; Gadea-Doménech and Espert-Tortajada, 2004; Gootjes et al., 2006; Hugdahl et al., 2009; Hiscock and Kinsbourne, 2011; Schmitz et al., 2018, for reviews and model explanations of the forced-attention REA). The theoretical background for this is that the FL-condition sets up a classic cognitive conflict (Miller and Cohen, 2001), not unlike the Stroop-situation (Stroop, 1935), where two conflicting stimuli, or components of a stimulus, are presented at the same time, and one of the stimuli, or components, is perceptually stronger and the other weaker. Considering the robustness of the NF REA it is surprising that it is so easily attenuated and even annihilated and shifted to an opposite LEA, just by instructing the subject to shift attention focus between the ears. An advantage of the forced-attention paradigm is that it is relatively simple to understand and execute. The only experimental manipulation between the FR- and FL-conditions is a single word “right” or “left,” all other parameters stay identical between the conditions, and still it has such profound effect on behavior.

Given that the basic REA is so easily modulated by changing the mindset of the subject, one would expect to find a similar modulation of brain activity when subjects move from the NF-condition to the FR-condition and especially to the FL-condition. Previous research in our own and collaborating laboratories using both PET and fMRI have shown that REA-related brain activity during the NF-condition in the temporal lobes in the vicinity of the auditory cortex and in the Wernicke area (BA 22), in the vicinity of the peri-Sylvian fissure, preferably, but not exclusively, on the left side (Hugdahl et al., 1999; Rimol et al., 2006; van den Noort et al., 2008). Against this background, we asked two questions in the present study, could we find a direct brain-behavior correspondence for the NF bottom-up condition by finding unique lateralized activations in the left hemisphere when directly contrasted with activations from homologous regions. This has been addressed in several studies before (e.g., van den Noort et al., 2008), but in these studies, there has not been a direct statistical comparison between homologous sites in the left and right hemispheres (but see Westerhausen et al., 2014). A second question raised was if there is a brain-behavior correspondence in that the profound change in behavior seen the FR- and FL-conditions compared to the NF-condition. In particular, we ask if there is a corresponding shift of activation from the temporal lobes to areas typically associated with higher cognition like attention and executive control functions. Most fMRI studies of attention have focused on visual attention and implicated the cingulate and parietal lobules (e.g., Badgaiyan and Posner, 1998; Coull and Nobre, 1998; Fan et al., 2005). However, since there is no reason to assume that attention should differ between sensory modalities, being a supra-modal cognitive function, we will assume that similar areas and networks would be implicated in the auditory modality. Such cross-over activation was shown by Cate et al. (2009) who found that auditory spatial attention activated visual areas. Similarly, executive and cognitive control functions have been shown to preferentially implicate dorsolateral prefrontal cortex and related regions along the midline, such as the anterior cingulate cortex (Braver and Cohen, 2000; Braver et al., 2002; Corbetta and Shulman, 2002).

These early findings with regard to brain areas implicated in higher cognition were later extended to the analysis of large-scale cortical networks subserving higher cognitive functions, such as the dorsal attention network (DAN) encompassing the intraparietal sulcus and the frontal eye fields, and the central executive network (CEN), broadly encompassing dorsolateral and medial frontal cortex (Bressler and Menon, 2010; Raichle, 2010; Petersen and Posner, 2012; Power and Petersen, 2013). These networks are often called task-positive networks because they are upregulated in situations with active task-processing and are also specific to the task or cognitive process implicated. In this respect, the DAN and CEN are in contrast to the default mode network (DMN) (Raichle et al., 2001) which is typically up-regulated in situations of rest, with no task present, and is therefore called a task-negative network. Hugdahl et al. (2015) suggested the existence of a third kind of network, in addition to task-negative (DMN) and specific task-positive (DAN, CEN) networks, which they labeled the extrinsic mode network (EMN) (Hugdahl et al., 2015). The EMN is suggested as a nonspecific task-positive network (see also Hugdahl et al., 2019; Riemer et al., 2020) which is upregulated across cognitive tasks and processes, being a generalized task-positive network. The EMN includes the supplementary motor area, anterior cingulate, lateral prefrontal cortex, and the inferior parietal lobule. The EMN shares properties with what Duncan (2013) labeled the attention demand system (see also Fedorenko et al., 2013), but is wider than an attention system. Hugdahl et al. (2015) proposed that the EMN is a brain network corresponding to requirements for mental flexibility and cognitive plasticity (cf. Blumstein and Amso, 2013). By bringing the findings from the forced-attention DL paradigm together with advances in functional neuroimaging we can now re-phrase the question of mind–brain correspondence with regard to auditory top-down modulation of bottom-up auditory laterality, and ask whether the forced-attention paradigm alters brain network connectivity in addition to changes in brain regions associated with attention and executive functioning.

## Methods

### Subjects

Data from two previous studies (van Wageningen et al., 2009; Dramsdahl, 2011) were anonymized, pooled and completely re-analyzed, and included 70 healthy adults who performed the forced-attention DL paradigm while in the MR scanner. There were 32 men and 38 women, all right-handed as determined from the Raczkowski et al. (1974) handedness questionnaire. Mean age for the whole sample was 27.23 (SD 6.04) years, 27.06 (SD 5.42) years for the men, and 27.37 (SD 6.59) years for the women. Since there were no significant interaction effects for sex, this factor is collapsed in subsequent analyses. The subjects had originally been recruited as healthy control subjects in two clinical projects; all were recruited from the Bergen metropolitan area between the years 2005 and 2010.

### Experimental Procedure

#### MR Scanning

Details of the MR scanning protocol and DL procedure have previously been described and presented in numerous publications from our laboratory over the years, see, for example, van Wageningen et al. (2009), Kompus et al. (2012), Westerhausen et al. (2014), and Hugdahl et al. (2019) for extended details. MR scanning was performed with a 3T GE Signa HDx scanner, using a single-channel head coil. Head movements were restrained by supportive padding, inside of the head coil. The functional imaging slices were positioned parallel to the AC-PC line, using a sparse sampling EPI acquisition protocol (cf. van den Noort et al., 2008), with the following parameters; 64 × 64 matrix, 25 slices, 5 mm slice thickness, 0.5 mm gap, TE 30 ms, TR 5.5 s. The TR was divided into 1.5 s TA and 4 s silent gap, when stimuli were presented and oral responses were recorded. Four dummy scans at the beginning of the scanning were discarded before analysis. Before the acquisition of the EPI-scans, a T1-weighted 3D volume image was acquired with a Fast Spoiled Gradient Recall sequence (FSPGR), with TE 14 ms, TR 400 ms, and IT 500 ms. This was used to acquire 188 sagittal slices covering the whole brain, with the following parameters; slice thickness 1 mm, no gap, matrix 256 × 256, FoV 256 × 256 mm^2^.

#### Dichotic Procedure

The dichotic experimental design and procedure was a classic session ON-OFF block-design with nine ON blocks, three per dichotic instruction condition (NF, FR, and FL). The nine ON-blocks started with the no-instruction condition (non-forced, NF), followed by either the forced-left (FL) or forced-right (FR) condition in a pseudo-randomized order. Each ON-block contained 10 EPI-volumes, a total of 55 s per block. Each ON-block was followed by a 55 s rest-condition without stimulus presentation, OFF-block. The paradigm consisted of dichotic presentations of pairs of CV-syllables, that is, two different syllables were simultaneously presented, one to the left and one to the right ear (Hugdahl and Andersson, 1986). The syllable pairs were formed combining the six syllables /ba/, /da/, /ga/, /pa/, /ta/, and /ka/ to obtain all possible 30 pairs of unidentical syllables (e.g., /ba/ presented to the left and /da/ presented to the right ear), that is, also including the reversed pairing (e.g., /da/ presented to the left and /ba/ presented to the right ear). The syllables were spoken by an adult Norwegian male voice with constant intensity and intonation. The syllables in each pair were temporally aligned to achieve simultaneous onset of the initial consonants. The stimulus duration varied between 400 and 450 ms. The first three ON-blocks were presented with no specific attention instruction (NF-condition), that is, the subjects were instructed to report the syllable which they heard best in each trial. For the remaining six blocks the subjects were asked to focus their attention on and report either the left-ear stimulus (FL condition) or the right-ear stimulus (FR-condition), respectively. In all three conditions, the instruction was to accurately report the syllable (with no emphasis on response speed). This approach of starting with the NF-blocks, followed by the forced-attention blocks, was chosen to avoid “carryover” effects that might result from presenting the forced attention conditions first since individuals might not be able to “not attend” once instructed to attend to a particular side in auditory space (Hiscock and Stewart, 1984). Before entering the MR-scanner all subjects conducted five practice trials (with the NF instruction) to familiarize them with stimulus material and procedure. Here, the subjects were also informed that in addition to the just-practiced NF-condition, two other conditions will be presented, during which they will be asked to selectively attend to one ear and only report the syllable presented to this ear. Inside the scanner, instructions were given via head-coil mounted goggles [NordicNeuroLab (NNL) Inc., Bergen, Norway, https://nordicneurolab.com/]. Each instruction consisted of a brief sentence asking the subject to orally report the syllable which was heard the best (NF), in the right ear (FR), in the left ear (FL), or to relax (rest block). The instruction screen was replaced after 2,500 ms by a fixation cross on which the subjects were instructed to focus their eyes. Stimulus administration was controlled by E-Prime software (Psychology Software Tools Inc., Pittsburgh, PA, https://pstnet.com/) and the dichotic stimuli were presented using MR-compatible headphones (NordicNeuroLab, Bergen, Norway, https://nordicneurolab.com/). The subjects' response was given orally and was recorded with an mp3-recorder connected to an MR-compatible microphone. The resulting recordings were later analyzed and coded. The percentage of correctly reported syllables was determined separately for the left-ear (LE) and the right-ear (RE) stimuli in each of the three ON-conditions.

### Data Processing and Statistical Analysis

The DL-data were statistically analyzed in a three-way ANOVA using the Statistica software (TIBC, USA, https://www.tibco.com/), with Ear (left, right) and Attention focus (NF, FR, FL) as within-factors. Significant interactions, and main-effects with more than two levels, were followed-up for separate contrasts between means, using Tukey's HSD test which controls for multiple tests. Effect-sizes for significant effects were calculated as partial eta-squared (η^2^). To test if the REA increased from the NF to the FR-condition, and decreased from the NF to the FL-condition, a laterality-index (LI) score was calculated for each condition separately, as [(RE – LE) / (RE + LE)] ^*^ 100. A positive LI score would indicate a REA, a negative value a LEA, and a zero value would indicate no-ear advantage (NEA). To evaluate changes in performance as a consequence of attention instruction, an attention gain-score (Westerhausen et al., 2015) was calculated, separately for gain in the FR- and FL-conditions. The gain-score was calculated as the increase of right-ear reports in the FR-condition relative to the right-ear reports in the NF-condition (FRRE–NFRE), and as the increase in left-ear reports in the FL-condition relative to the left-ear reports in the NF-condition (FLLE–NFLE). The gain-scores were subjected to one-sample t-tests, separately for the FR and FL gain-scores.

fMRI-data were first pre-processed and analyzed with the SPM12 software package (https://www.fil.ion.ucl.ac.uk/spm/), which runs under MATLAB (MathWorks Inc., Natick, MA, USA), applying standard SPM parameter settings (following the routine used by Hugdahl et al., 2019). This included the following steps; the DICOM images were converted to nifti-format, and pre-processed following SPM routines for realignment, unwarping, and normalizing the EPI-images to the MNI-template, and smoothed with an 8-mm kernel, and with a high-pass filter of 512 sec. The individual EPI-images were then subjected to a SPM first-level analysis where significant regions were determined using the t-statistics. The resulting contrast images were then used as input for a SPM second-level analysis in a one-way ANOVA design. These analyses involved comparing first the three attention-instruction conditions separately against the OFF-blocks, and then the comparison of the FR- and FL-conditions against the NF-condition, and between the FR-condition against the FL-condition, and vice versa. This last comparison was done to reveal any differences in brain activation between the two directed-attention (FR- and FL-) conditions. If not otherwise specified all comparisons were made with a significance threshold of 0.05, FWE corrected, and with a minimum of 10 voxels to identify a cluster. A second region-analysis involved a left-right laterality comparison where the EPI-images were flipped across the axial mid-line (Friston, 2003; Westerhausen et al., 2014), using t-test. This allowed for a direct left-right comparison of homologous voxels to reveal a basic underlying auditory laterality effect to dichotic CV-syllable stimulation. Image flipping was performed with in-house scripts which loaded the standard-space EPI data using SPM functionality (spm_read_vols), and flipped volumetric data in the X-axis using the MATLAB “flipdim” function before writing to a new output file.

The SPM region analysis was complemented with a network analysis, focusing on connectivity, using the CONN v 19.c toolbox software (Whitfield-Gabrieli and Nieto-Castanon, 2012), which also runs under MATLAB 2021a. Functional imaging data were pre-processed using the CONN default pre-processing pipeline for volume-based analyses. The steps for functional data comprise realignment and unwarping for subject motion estimation and correction (12 parameters). Next, centering to (0,0,0) coordinates and ART-based outlier detection identification was applied. Segmentation and normalization to MNI space were applied next. As a last step, we applied smoothing using spatial convolution with Gaussian kernel of 8 mm FWHM. Structural data were translated to (0,0,0) center coordinates and segmented (gray/white/CSF) and normalized to MNI-space. In the denoising step, we applied band-pass filtering (0.002–0.09 Hz) and regression of realignment parameters (12), white and gray matter, and CSF confounds. For the seed-based functional connectivity analyses, we used the default atlas implemented in CONN. This atlas includes 132 regions from the FSL Harvard-Oxford atlas and AAL atlas, with an additional atlas of commonly used networks and areas (defined from CONN's ICA analyses of Human Connectome Project dataset).

#### First-Level Analysis

Functional connectivity (FC) measures were computed using pre-defined seed regions and networks. Computation of bivariate Pearson correlations between the extracted mean Blood-Oxygenation-Level-Dependent (BOLD), signal time-courses of region-of-interests (ROIs) allowed to identify patterns of condition-based functional connectivity. Before the second-level analyses, all the connectivity measures were normalized using Fisher's transformation to improve subsequent second-level analyses and the distribution of the scores.

#### Second-Level Analysis

For group-level results, we calculated ROI-to-ROI connectivity correlations, threshold with an FDR corrected *p* < 0.05. Each condition was set as a separate contrast (NF, FR, FL, and OFF) separately. This was the basis for connectivity rings (see **Figure 4**). To achieve a better overview of the results, ROIs were sorted using hierarchical clustering and CONN networks a priori order (32 regions compromising 8 networks). Statistical significance check followed standard settings for cluster-based interferences (FDR corrected at *p* < 0.005).

## Results

### Behavioral Results

**Figure 1:**
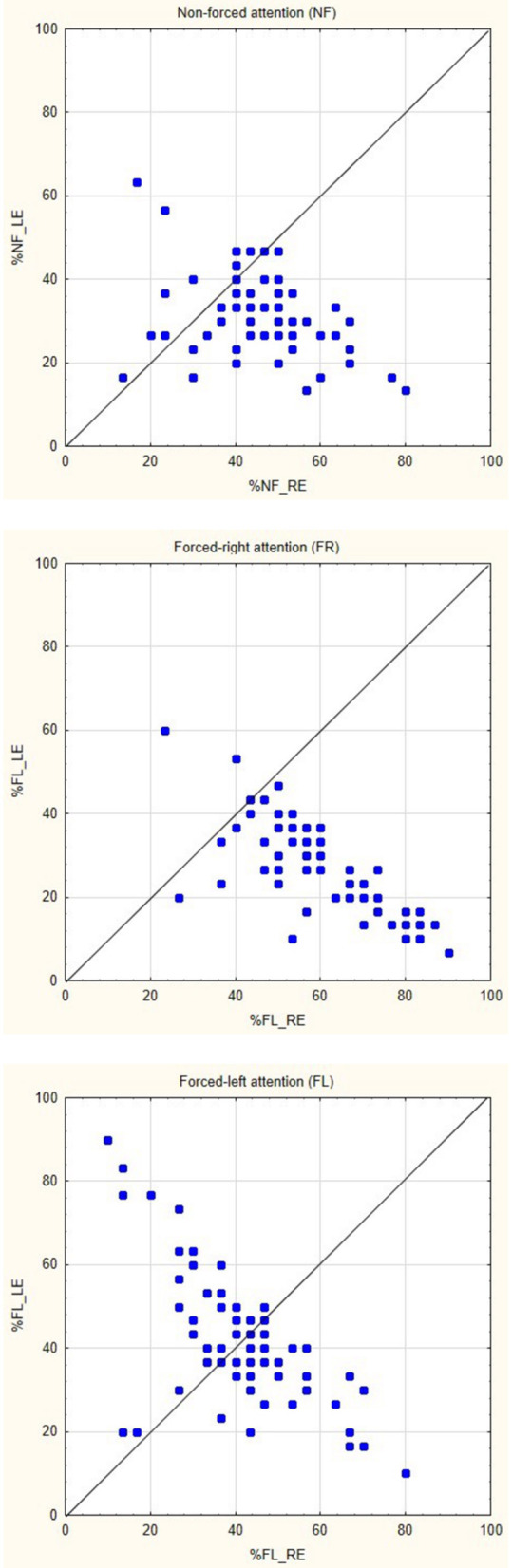
Scatter-plots of individual percentage scores (0–100) for the right (RE) and left (LE) ear scores, split for the three attention instruction conditions non-forced (NF), forced-right (FR), and forced-left (FL).

The ANOVA showed significant main-effects of the within-effects factors ear and attention, *F*_(1,68)_ = 59.694, *p* < 0.000001, η^2^ = 0.467 and *F*_(2,136)_ = 13.111, *p* < 0.00001, η^2^ = 0.162, respectively. The interaction of ear × attention was also significant, *F*_(2,136)_ = 45.692, *p* < 0.000001, η^2^ = 0.402. The interaction revealed a significant REA in the NF and FR conditions, and NEA in the FL-condition, when tested with Tukey's HDS test (*p* < 0.05 for the significant comparisons). See Table 1 for mean percentage of correct reports separated for the right and left ear scores, for the NF, FR, and FL attention conditions, and Figure 1 for corresponding scatter plots for the three attention conditions, which show the actual distributions of individual scores across conditions. There was in addition a borderline significance for the main-effect of sex, *F*_(1,68)_ = 3.848, *p* = 0.054, with a very small effect-size, η^2^ = 0.053. Females had overall, across all factors, slightly higher mean correct reports compared to males (Means = 42.383, SD 4.623 and 40.208, SD 4.621). No interaction with the factor sex was significant.

**Table 1:** Mean and SD percentage scores for the right (RE) and left (LE) ear split for the three attention instruction conditions (NF, FR, and FL).

**Variable**	**Valid N**	**Mean**	**Std. Dev**.
%NF_RE	70.00	46.71	12.80
%NF_LE	70.00	32.10	9.65
%FR_RE	70.00	59.33	14.14
%FR_LE	70.00	27.05	10.95
%FL_RE	70.00	41.67	14.42
%FL_LE	70.00	41.48	16.18

The laterality index (LI) scores were subjected to a repeated- measures ANOVA with the three LI scores for each subject as input. This showed a significant main-effect of the attention focus instruction, *F*_(2,138)_ = 46.899, *p* < 0.000001, η^2^ = 0.405. Follow-up tests with Tukey's HSD test showed that the FR REA was significantly larger than both the NF REA and the FL NEA. In addition, the NF REA was significantly larger than the FL NEA (all contrasts, *p* < 0.05). The means for the three LIs were, NF: 17.712, SD 22.54, FR: 36.736, SD 25.32, FL: 0.808, and SD 31.18, respectively. The analysis of the gain-scores showed that both the FR and FL gain-scores were significantly different from the NF baseline scores, *t*_(70)_ = 12.619, *p* < 0.000001, and 9.380, *p* < 0.000002, respectively. The gain-score results mean that there was a significant gain in right-ear scores in the FR-condition relative to the right-ear NF-condition, and a corresponding gain in left-ear scores in the FL-condition relative to the left-ear NF-condition (see Table 1).

### fMRI Results

Functional MRI data are first presented as comparisons between the three attention instruction conditions, using a standard SPM region approach. This approach also included a direct comparison of activation between the left and right superior temporal gyrus (STG) region by flipping the axial images along the midline along the *x*-axis. This was done to statistically evaluate a left-sided laterality effect for phonetic sounds. The SPM approach was followed by a cortical network connectivity approach using graph theory in the CONN toolbox software.

#### SPM Region Approach

To achieve an overview of activations, later to be contrasted against each other, we first analyzed each instruction condition separately. For the NF-condition, the SPM analysis showed significant activations in the left and right superior temporal gyrus (STG), extending into the middle temporal (MTG), Heschl's gyri (HG), and planum temporale (PT) region, with peak x, y, z mm coordinates at −58, −18, 2 and 58, −16, −4 for the left and right activations, respectively. The corresponding activations for the FR-condition were essentially similar, with peak x, y, z coordinates at −58, −20, 2 for the left, and 58, −16, −4 for the right STG/MTG/planum temporale (PT) region. The FR-condition revealed, in addition, significant activations bilaterally in the supplementary motor area (SMA) with peak coordinates x −2, y 4, z 54, in the right pallidum with the corresponding peak coordinates 20, −2, −8, and in the left lingual gyrus (LiG) with peak coordinates x −28, y −60, z −2. The FL-condition showed significant activations in the left and right STG/MTG similar as for the NF- and FR-conditions, peak coordinates being x −58, y −20, z 2, and 54, −18, −4, respectively. The FL-condition, in addition, showed significant activations bilaterally in the SMA, peak coordinates x 0 y 2 z 56, thalamus with peak coordinates x −12, y −18, z −2 and x 12, y −16, z 2, left and right, respectively, left middle frontal gyrus with peak coordinates at x −36, y 42, z 30, and in the right cerebellum with peak coordinates at x 14, y −64, z −28.

**Figure 2:**
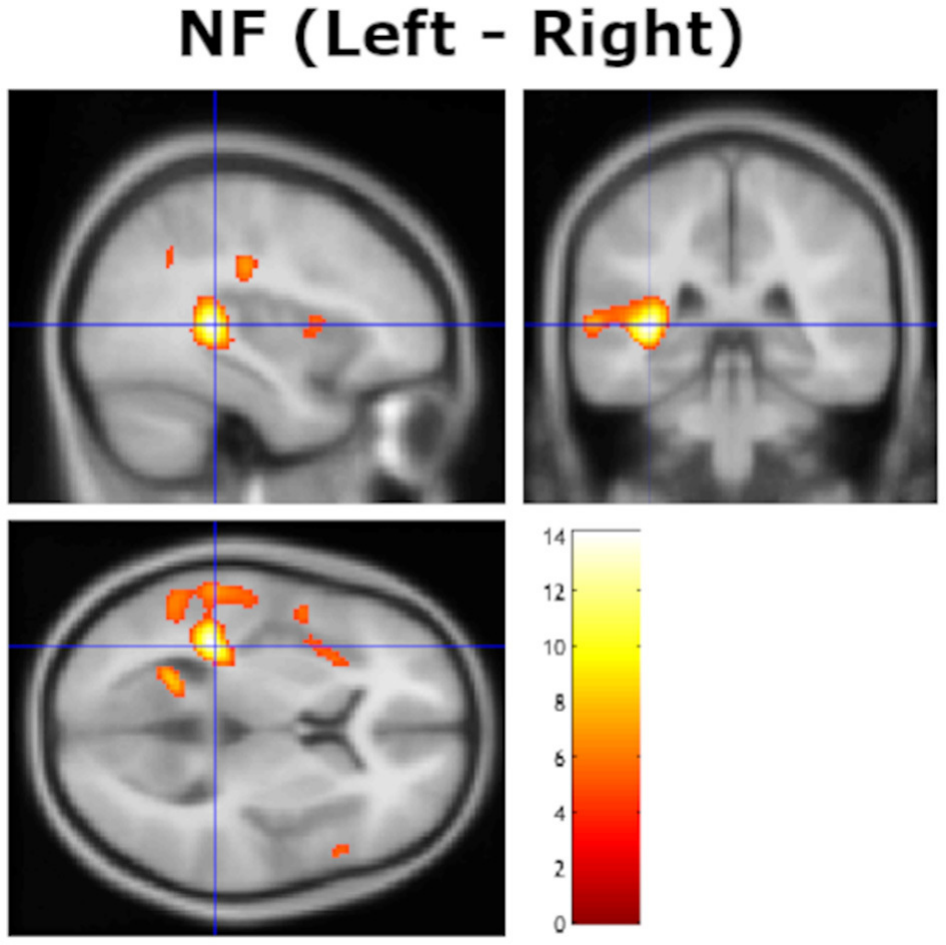
Remaining significant activation in the left hemisphere after contrasting with homologous voxels in the right hemisphere for the non-forced (NF) attention instruction condition, thresholded at *p* = 0.05 (FWE corrected). The cross-hair is at *x, y, z* coordinates −36, −33, 9. Color bar shows *t*-values.

A second analysis evaluated bilateral STG/MTG/Heschl's gyri activations, with a hypothesis that it would be stronger on the left side, showing a left hemisphere laterality effect that would correspond to the behavioral NF REA (cf. Hugdahl et al., 1999; van den Noort et al., 2008). This was done by contrasting homologous images on the left and right side, when images were “flipped” in the X-direction along the midline (cf. Friston, 2003). The statistical analysis showed a significant remaining left-over-right activation in the planum temporale (PT), and in Heschl's gyrus with peak x, y, z coordinates at −36, −36, 6, in the left post-central gyri with peak x, y, z coordinates at −48, −20, 38, and in the left precuneus with peak x, y, z coordinates at −22, −56, 10 (see Figure 2).

**Figure 3:**
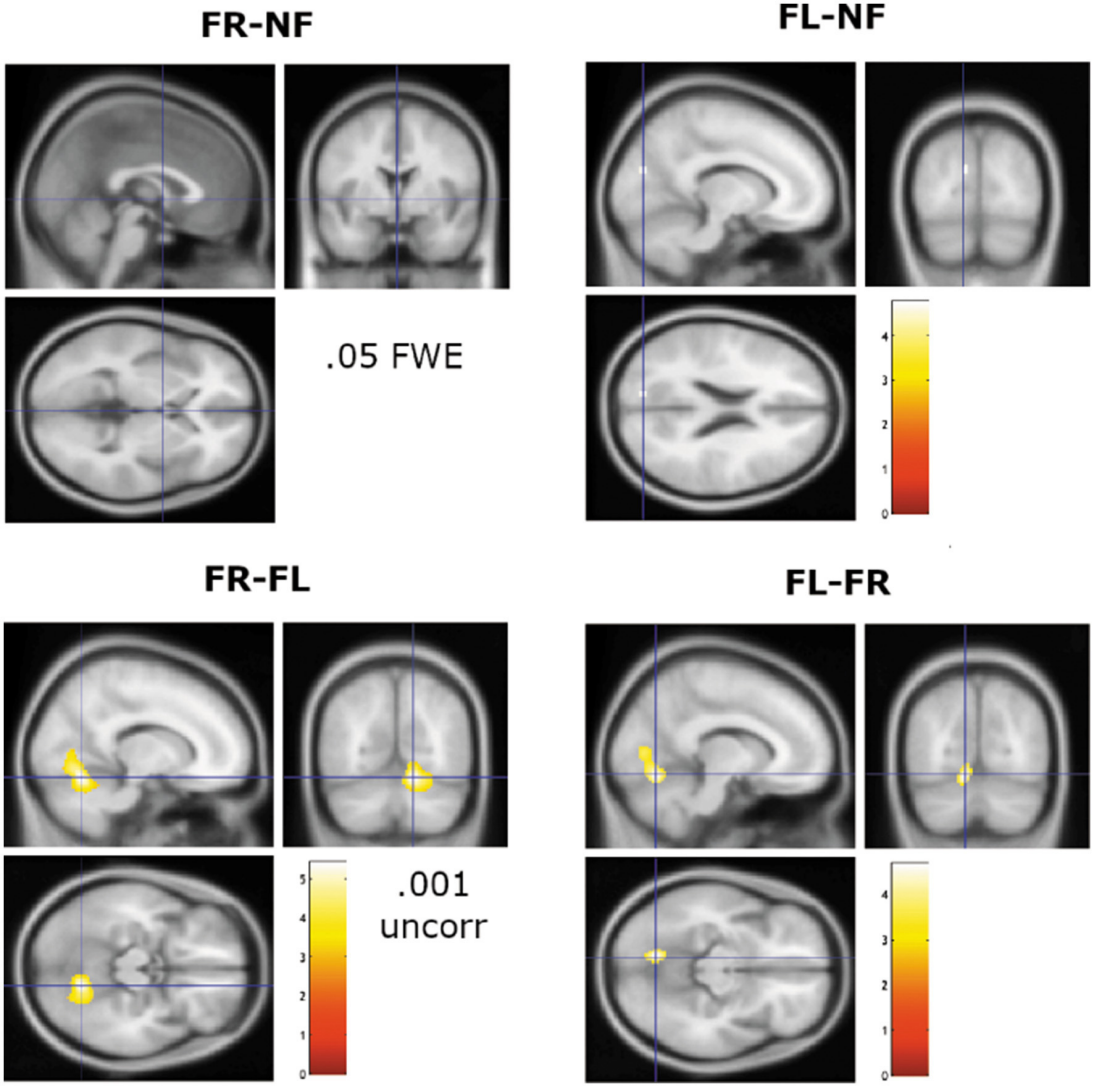
Remaining significant activations when contrasting the FR and FL attention instructions against the NF baseline condition, and against each other. Note lowering of significance threshold to *p* = 0.001 (uncorrected) for the sections shown in the lower panel. Cross-hair is at *x, y, z* coordinates 0, 0, 0 mm for the FR-NF contrast and at −12, −80, 22 mm for the FL–NF contrast. For the lower panel, the cross-hairs are placed at 14, −64, −16 mm for the FR–FL contrast and at −10, −70, −12 mm for the FL-FR contrast. Color bar shows *t*-values.

A third region-analysis was for contrasting the three attention instruction conditions (NF, FR, and FL) against each other. To highlight the focus on additional activations in the FR- and FL-conditions above and beyond activations observed in the NF-condition, we here report only the subtractions for FR and FL with the NF-condition, and the subtraction of FR against FL, and vice versa (see Figure 3).

The FR–NF contrast did not yield any surviving significant results with a 0.05 FWE corrected significance threshold. The FL–NF contrast yielded significant remaining activations in the left cuneus/precuneus with peak x, y, z coordinates at −12, −80, 22. The next comparisons were for the FR–FL and FL–FR contrasts, which showed no remaining activations at FEW 0.05 corrected significance threshold. Lowering the significance threshold to 0.001 uncorrected and with a cluster size to 10 voxels, revealed unique activations for the FL–FR contrast in the left lingual gyrus, extending into the left calcarine cortex, with peak x, y, z coordinates at −10, −70, −12 and −8, −76, 6. The reverse contrast, FR–FL yielded a significant cluster in the right lingual gyrus with peak x, y, z coordinates at 14, −64, −16, extending into the central operculum with peak x, y, z coordinates at −38, −20, 20. There was also a small significant cluster in the vicinity of the left triangular part of the inferior frontal gyrus, and left middle frontal gyrus, with peak x, y, z coordinates at −44, 36, 12.

#### CONN Network Approach

**Figure 4:**
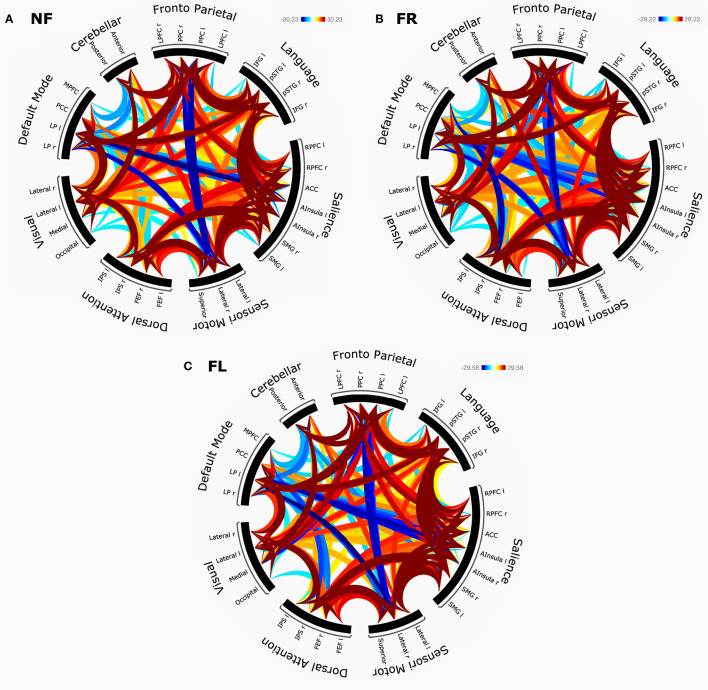
**(A–C)** The panel shows corresponding network connectivity rings for eight cortical networks, split for NF (**A**, top left), FR (**B**, top right), FL (**C**, bottom) attention instruction condition.

The results of the network connectivity analyses based on graph theory and seed-correlations are shown in Figures 4A–C with separate connectivity rings for the NF-, FR-, and FL-conditions.

The results of these analyses based on graph theory and seed-correlations are shown in Figures 4A–C with separate connectivity rings for the NF (top), FR (middle), and FL (bottom). All graphs were FDR corrected at *p* < 0.005.

For the non-forced (NF) condition (Figure 4A), there were statistically significant negative correlations between the salience and default mode networks, in particular between the anterior cingulate cortex (ACC) and left and right lateral parietal (LP) nodes of the two networks. Negative correlations were also shown between the left lateral prefrontal cortex (LPFC) and precuneus cortex (PCC) nodes of the fronto-parietal network and the sensori-motor network, and between the left and right LP nodes of the default mode network and sensori-motor network areas. As expected for the DL paradigm, we observed positive correlations between the language network and the fronto-parietal network. The salience network was highly positively correlated with the language network, especially with the left and right superior temporal gyrus (STG) and left and right inferior frontal gyrus (IFG) nodes of the two networks. This was also in line with the findings presented for the SPM analysis.

For the forced right (FR) condition (Figure 4B), there was again a high positive correlation between the salience and language networks, especially for the left and right STG and IFG nodes, and with the left and right intra-parietal sulcus (IPS) nodes of the dorsal attention network. As for the NF-condition, the ACC and portions of the anterior insula nodes of the salience network were negatively correlated with the DMN. The same region was however positively correlated with the left STG node of the language network, and with the LPFC and PPC nodes of the fronto-parietal network. As expected, we observed a negative correlation between the medial prefrontal cortex (MPFC) node of the DMN and the left and right IPS nodes of the dorsal attention network. The left and right STG were in addition positively correlated with the left and right LP nodes of the DMN. Finally, there were positive correlations between the fronto-parietal network and the LP node of the default mode network.

For the forced-left (FL) condition (Figure 4C) there were strong negative intrinsic correlations between the left and right LP nodes of the DMN. There were negative correlations between the ACC and portions of the anterior insula nodes of the salience network, on the one hand, and the DMN on the other hand. This was seen in all three instruction conditions. Negative correlations were also seen between the DMN and the dorsal attention network, again across all three instruction conditions. The FL-condition is cognitively the most demanding condition. Therefore, the ACC node of the salience network was strongly positively correlated with the STG nodes of the language network, and within itself. Positive within-network correlations were also found between the left and right insula with rostral prefrontal cortex (RPFC) of the salience network. The right anterior insula and right supra-marginal gyrus (rSMG) yielded positive interaction with the left portion of intra-parietal sulcus in the dorsal attention network. Interestingly, default mode network revealed a positive correlation with fronto-parietal network, left and right lateral prefrontal cortex (LPFC) and (PCC).

There were no significant differences between the three conditions when directly comparing the connectivity patterns across conditions. There were some differences in the large *t*-test matrix produced when comparing nodes across networks, but these were probably spurious effects and we have chosen to not report them as reliable findings.

## Discussion

Starting with the behavioral data, the present study replicated numerous previous findings of a significant REA in adult right-handed individuals, originally reported by Kimura (1961) and Bryden (1963) (see Jäncke and Shah, 2002; Tervaniemi and Hugdahl, 2003; Hugdahl and Westerhausen, 2016; for reviews, Westerhausen, 2019). The current results are also in line with previous electrophysiological and hemodynamic imaging studies by showing a neuronal locus for the non-forced REA in the left posterior temporal lobe (Hugdahl et al., 1999; Brancucci et al., 2004, 2008; van den Noort et al., 2008).

The current study extends previous functional imaging studies by providing direct statistical evidence of increased activation in the left peri-Sylvian region when contrasted against the homologous right side for each subject [but see Westerhausen et al. (2014) who applied a similar technique when contrasting homologous sites across the axial midline]. In a similar way, the current study replicates previous studies of modulation of the REA when instructing the subject to actively attend to and report only the right or left ear stimulus, which was labeled the “forced-attention” effect by Hugdahl and Andersson (1986), see also Bryden et al. (1983), Westerhausen and Hugdahl (2010) and Hiscock and Kinsbourne (2011) for reviews. As shown in Figure 1, there was a clear behavioral effect of inducing an attentional bias in favor of either the right or left ear, with an expected increase in the magnitude of the REA in the FR-condition compared to the NF baseline condition, and an expected decrease in the FL-condition. There was essentially no discernible difference for the right and left ear scores in the FL-condition, as depicted in Table 1, with a 5% decrease in the right ear score and about 10% increase in the left ear score compared to the NF baseline condition. In this respect, the current results follow numerous previous studies when it comes to attentional modulation of the behavioral ear advantage (see Westerhausen and Hugdahl, 2010; Hiscock and Kinsbourne, 2011, for reviews). The basic behavioral REA in the NF attention instruction condition was replicated in the brain activation data with unique left hemisphere activation in the STG/HG region not seen on the right side after contrasting the left with the right-side activation in a direct statistical test, by “flipping the images” (see Figure 2).

However, the rather dramatic behavioral effects associated with varying instruction of which ear to attend to as shown in Figure 1 did not show up in corresponding dramatic differences in neuronal activation, neither in the regional nor in the network connectivity analysis (see Figures 3, 4). This was somewhat unexpected since previous studies have shown differences in activation patterns between the FR- and FL-conditions. The first hemodynamic imaging publications on the effects of attention bias in DV to syllables were the studies by O'Leary et al. (1996) and Hugdahl et al. (2000), being PET-studies. Both studies reported effects of focusing attention, but none of the studies did a direct comparison between FR- and FL-conditions, and O'Leary et al. (1996) in addition used CV-syllables as stimuli, while Hugdahl et al. (2000) used CV-syllables. It should also be mentioned that the samples were quite small, O'Leary et al. (1996) had 10 and Hugdahl et al. (2000) had 12 subjects included. Over the years since the original publications, there have been several other studies investigating neuronal effects of the dichotic “forced-attention” paradigm. These have shown various findings not easily related to the behavioral effects. For example, Kompus et al. (2012) found unique activations in the left inferior prefrontal cortex and caudate nucleus in the FL-condition using fMRI. This was not found by Alho et al. (2012) who reported that focusing attention to either the right or left ear resulted in stronger neuronal activity in the contralateral left or right temporal cortex (see also Jäncke et al., 2001). Alho et al. (2012) used MEG while Kompus et al. (2012) used fMRI which could partly explain the differences, but only partly since it would mean that any differences are dependent on the measure used. Adding to this, Eskicioglu et al. (2019) found increased hemodynamic responses in the FR- and FL-conditions compared to a NF-condition, but with no difference between the two attention conditions. Finally, Thomsen et al. (2004b) found no uniquely activated areas when comparing the FL with the FR attention instruction conditions. Thomsen et al. (2004b) therefore concluded that the overall increase in activation seen in the prefrontal cortex in the FR- and FL-conditions relative to the NF-condition was more related to stimulus discrimination that to cognitive load. Again, the sample sizes were quite small, Jäncke et al. (2001) had 11 subjects, while Alho et al. (2012) reported data from 15 subjects, and Eskicioglu et al. (2019) had 26 subjects. In addition to a larger sample size, the present study also adds to previous imaging studies by including a network connectivity analysis supplementing a traditional region approach.

For both analysis approaches, we did not find any clear unique brain markers of differences between conditions, especially not for the FR- and FL-conditions. The SPM region-approach showed essentially similar brain activation patterns across all three attention instruction conditions (NF, FR, and FL) when applying a strict significance threshold (*p* < 0.05 FWE corrected). Lowering the threshold to 0.001 uncorrected for the higher-order FR–FL and FL–FR contrasts showed unique remaining activations in both anterior and posterior brain regions, primarily in the cuneus/precuneus region and in frontal and insular regions. In this respect the present findings are in line with the results reported by Kompus et al. (2012) who also had a reasonably large sample of 113 subjects. An interesting difference between the FR- and FL-conditions was the lingual gyrus/operculum activations, with dominance on the left side for the FL–FR comparison, but with a right-side dominance for the FR–FL comparison. It should be remembered however that these findings occurred only after lowering the significance threshold, which is a questionable procedure. A first conclusion is therefore that the large and statistically robust behavioral effects seen in the “forced-attention” DL paradigm, so labeled by Hugdahl and Andersson (1986) when manipulating focus of attention, do not have corresponding brain markers. Statistically speaking, the connectivity analysis showed a similar pattern, with basically no difference in network connectivity between the three attention focus conditions, as seen in Figures 4A–C. However, the connectivity-rings shown in Figures 4A–C revealed strong positive bilateral correlations between the language and salience, fronto/parietal and dorsal attention networks, between the dorsal attention and sensory/motor networks, and between the fronto parietal and default mode network, to mention some of the more prominent connections.

A question that arises is how the present findings relate to the classic theoretical models of Kimura (1967) and Kinsbourne (1970) models of DL. Kimura's structural model assumes that during auditory input competition, the signal contralateral to the left, language, hemisphere prevails over the ipsilateral signal. This is so because it blocks the left ear ipsilateral signal from direct input to the speech processing regions in the left hemisphere, which causes the REA. The Kimura model makes no assumptions about the effect of focusing attention to the left or right, nor regarding bottom-up versus top-down effects. It only predicts a REA in the non-forced situation, which both the behavioral and the SPM region-approach support. The direct contrast of activation between the left and right STG/HG/Planum temporale region showed remaining activation only on the left side, supporting a view of neurons in this region to be sensitive to dichotic presentations of verbal input. Kinsbourne's attentional model assumes that the REA is attributed to a dynamic imbalance in the activation of the cerebral hemispheres, resulting in an attentional bias to either the left or right side. Dichotically presented verbal input will activate the left hemisphere more than the right hemisphere as outlined in Hiscock and Kinsbourne (2011). The result is a right-sided bias of attention which causes the behavioral REA. An unanswered question in Kinsbourne's model is why a bias of attention should automatically follow a bias in hemisphere activation, with no instruction to focus attention to either side. The present results find no support for such a view since there was no unique activation of the attention network in the NF-condition, nor of differential attention network activation in the FR- and FL-conditions, which the model would predict. The present results are therefore better explained with reference to Hugdahl's model (Hugdahl et al., 2009) of an interaction between bottom-up (stimulus-driven) and top-down (instruction-driven) effects on the modulation of the REA in the “forced-attention” paradigm.

We predicted that the EMN should be negatively, or anti-, correlated with the DMN, and looking at Figures 4A–C it seems that there are anti-correlations between the DMN on the one hand and in particular, the salience and dorsal attention networks on the other hand, which would be expected, since these networks overlap with the EMN to a large extent (see Hugdahl et al., 2019; Riemer et al., 2020). The pattern of these anti-correlations seems however to be equally distributed across the three attention instruction conditions, which was not predicted. If it is acknowledged that all three conditions require a minimum of cognitive resources for effective processing, failure of finding unique brain markers for each condition may be explained within an EMN network perspective. In their 2015 paper, Hugdahl et al. suggested the EMN “as an umbrella term for all these networks that share a common activation pattern structure, and [which are] up-regulated during task processing, but independent of the specific cognitive task-structure” (pp. 4–5). It is interesting to note that what these authors meant by “all these networks” were exactly what have been identified as the salience, dorsal attention and fronto-parietal networks in the current study. The core nodes of the EMN, found across a range of cognitive tasks and processes, are including the inferior and middle frontal gyri, inferior parietal lobule, supplementary motor area, and the inferior temporal gyrus. These areas overlap with areas found with significant connections in the connectivity analysis. Thus, a reason why a specific pattern of activated regions or networks emerge when the subjects move through the three attention instructions, and especially why we fail to see this for the FR and FL comparison, may be that these tasks engage the EMN network which is non-specific to the task and processing demands. In other words, the EMN would be equally up-regulated in the FR- and FL-condition, which is what the present results have shown.

A final word on the combination of traditional SPM region-analysis and CONN network analysis approach. Combining such approaches may be a better solution than only reporting from one approach since they focus on different aspects of fMRI data. To use a metaphor: a region approach identifies unique trees in the forest, but fails to see the forest, a connectivity approach identifies the forest but fails to see unique tress. Combining the two may therefore increase resolution and circumvent limitations imposed by the approaches in isolation.

To sum up the main findings, we replicated previous behavioral findings of a REA in the NF attention instruction condition and showed that this has a direct left hemisphere laterality basis, with unique brain activation in the left STG/HG region when contrasting the two sides across the axial midline, by “flipping” the images (Friston, 2003). In this respect, there were unique brain markers for the NF behavioral laterality effect. When it came to the FR and FL attention instructions, we failed to see unique brain activation patterns matching the unique behavioral effects seen for these conditions. For both the SPM region-approach and the CONN connectivity-approach, there were predicted network connections, both negative and positive connections. There were, however, no significant differences between the instruction conditions, which were conceptualized within an EMN network theoretical frame.

## Data Availability Statement

The raw data supporting the conclusions of this article will be made available by the authors, without undue reservation.

## Ethics Statement

The studies where the data were taken from were reviewed and approved by Regional Committee for Medical Research Ethics in Western Norway. The patients/participants provided their written informed consent to participate in these studies. Data had been anonymized before being entered into the current study.

## Author Contributions

KK analyzed the data and wrote the manuscript. AC, KS, and LS analyzed the data and commented on the manuscript. LE contributed data acquisition and commented on the manuscript. MD commented on the manuscript. KH designed the study, analyzed the data, and wrote the manuscript. All authors contributed to the article and approved the submitted version.

## Funding

This study was funded by ERC Advanced Grant to KH #693124.

## Conflict of Interest

KH, LE, KS, and AC have shares in the NordicNeuroLab Inc. company that produced add-on equipment for the fMRI acquisition. The remaining authors declare that the research was conducted in the absence of any commercial or financial relationships that could be construed as a potential conflict of interest.

## Publisher's Note

All claims expressed in this article are solely those of the authors and do not necessarily represent those of their affiliated organizations, or those of the publisher, the editors and the reviewers. Any product that may be evaluated in this article, or claim that may be made by its manufacturer, is not guaranteed or endorsed by the publisher.
